# Double-Cone Coil TMS Stimulation of the Medial Cortex Inhibits Central Pain Habituation

**DOI:** 10.1371/journal.pone.0128765

**Published:** 2015-06-05

**Authors:** Federico D’Agata, Alessandro Cicerale, Arianna Mingolla, Paola Caroppo, Laura Orsi, Paolo Mortara, Walter Troni, Lorenzo Pinessi

**Affiliations:** 1 LabNI, University of Turin, Turin, Italy; 2 Neuroscience Department, University of Turin, Turin, Italy; 3 Institut du Cerveau et de la Moelle épinière, ICM, Hôpital Pitié Salpêtrière, Paris, France; 4 Neuroscience Department, AOU Citta della Salute e della Scienza (Presidio Molinette), Turin, Italy; University Medical Center Goettingen, GERMANY

## Abstract

**Objective:**

The aim of this study was to investigate whether Transcranial Magnetic Stimulation (TMS) applied over the medial line of the scalp affects the subjective perception of continuous pain induced by means of electric stimulation. In addition, we wanted to identify the point of stimulation where this effect was maximum.

**Methods:**

Superficial electrical stimulation was used to induce continuous pain on the dominant hand. At the beginning of the experiment we reached a pain rating of 5 on an 11-point numeric rating scale (NRS; 0 = no pain and 10 = maximum tolerable pain) for each subject by setting individually the current intensity. The TMS (five pulses at increasing intensities) was applied on 5 equidistant points (one per session) over the medial line of the scalp in 13 healthy volunteers using a double-cone coil to stimulate underlying parts of the brain cortex. In every experimental session the painful stimulation lasted 45 minutes, during which pain and distress intensities NRS were recorded continuously. We calculated the effect of adaptation and the immediate effect of the TMS stimulation for all locations. Additionally, an ALE (Activation Likelihood Estimation) meta-analysis was performed to compare our results with the neuroimaging literature on subjective pain rating.

**Results:**

TMS stimulation temporarily decreased the pain ratings, and pain adaptation was suppressed when applying the TMS over the FCz site on the scalp. No effect was found for distress ratings.

**Conclusions:**

The present data suggest that the medial cortex in proximity of the cingulated gyrus has a causal role in adaptation mechanisms and in processing ongoing pain and subjective sensation of pain intensity.

## Introduction

Pain, as defined by International Association for the Study of Pain (2011), is “an unpleasant sensory and emotional experience associated with actual or potential tissue damage, or described in terms of such damage”. Beyond the peripheral components of the nociceptive system lies a network of brain areas, dubbed the pain matrix [1], that elaborates sensory and biochemical inputs and produces the perception of pain. The lateral structures of this network are thought to have a sensory function and therefore code intensity and spatial localization of pain, while medial areas (such as the anterior (ACC) and (MCC) middle cingulate cortex) play a cognitive, attentive and emotional role [2,3]. The mechanisms that generate the experience of pain can be inhibited (antinociception) or facilitated (pronociception) [3,4] by the descending pain modulatory system. This system is constituted by a network of areas such as the ACC, the insulae, the amygdalae, the periaqueductal gray (PAG) and the rostral ventromedial medulla [4]. In particular, the antinociceptive component of the pain modulatory system, which counts the cingulate cortex among its components, is responsible for opiate analgesia and also for effects such as placebo analgesia or the inhibition of pain during a fight or flee response [5]. In particular, the ACC and the anterior part of the MCC are involved in almost all phenomena related to the antinociception. For instance, a functional imaging study [6] showed that the ACC activates both after administration of exogenous opioids and in conditions where the subjects were given a placebo, showing a link between placebo analgesia and the opioid system, while some studies [6,7] have strengthened the evidence supporting the importance of the rostral ACC in analgesia and its connection with other areas of the antinociceptive network (amygdalae and PAG). The ACC also seems to be linked to adaptation mechanisms: it was shown [8] that the subgenual ACC (sgACC) plays a role in the habituation to painful stimuli administered during several days, while other authors [9] suggested that the sexual differences in the connectivity of the sgACC with the antinociceptive system can at least in part explain the higher level of pain adaptation shown by woman compared to men. The role of the ACC in habituation is confirmed by [10], as the authors found that the rostral ACC and the PAG were the only areas of the pain matrix whose activity increased more in subjects with quicker physiological (electrodermal activity, EDA) habituation than in subjects with a slower EDA during a prolonged painful stimulus. They conclude that functional activation in these areas reflects an antinociceptive process that could mediate habituation in the other areas of the pain matrix.

As it seems clear that the rostral cingulate cortex is involved in pain adaptation, it is interesting to investigate whether this phenomenon can be modulated by noninvasive stimulation. It is known that pain can be modulated by both invasive [11] and noninvasive [12] brain stimulation. There are few accounts of the stimulation of the ACC using TMS, and this might be partly explained by the fact that the ACC (as other relatively profound brain structures) is not easily reachable using a figure of eight coil stimulation [13]. Some studies tried to stimulate the medial frontal cortex (MFC), which lies immediately above the anterior part of the MCC, and the results were contradictory. One of the first studies to investigate the effect of TMS on nociception [14] used pairs of TMS pulses (ppTMS) to disrupt the activity of areas known to be involved in pain processing. The authors found that while stimulation of the primary sensorimotor area (SMI) has a pronociceptive effect, the stimulation of the MFC seems to be antinociceptive. This is in contrast with the majority of recent studies [12], but shows a dissociation between the effect of the stimulation of the MFC and of SMI, which recent studies have found [12,15]. Stimulation of the MFC has been shown to increase the perception of pain: [16] applied ppTMS over the MFC and found that when the pulses are applied shortly (25–75 ms) after the painful stimulation, ppTMS can enhance the sensation of pain. The authors interpreted it as an effect of the interference between the magnetic pulses and the nociceptive input, and [15] found that high frequency repetitive TMS (rTMS) on MI increased sensory perception and pain tolerance thresholds. On the contrary, the pain tolerance threshold was decreased after the application of the rTMS over the MFC, but not the sensory perception. The heterogeneity in the results could be explained by noting that the temporal distance between the painful stimulus and TMS pulses, as well as rTMS frequency, can affect the magnitude and the direction (excitatory or inhibitory) of the effect on behavior [17,18]: therefore, applying different stimulation protocols can obtain different results.

In this study, we adopted a protocol of TMS stimulation similar to the ones that have been shown to disrupt the activity of the targeted brain area. In order to be able to stimulate deeper brain structures (ACC and MCC) we used a double-cone coil [19]. We tested the effect of the TMS stimulation on various points along the sagittal midline of the brain (from AFz to CPz in EEG 10–10 system) to assess the different roles played by different parts of the cingulate cortex in pain processing. To be able to study habituation mechanisms we adopted a continuous pain paradigm, recording pain and distress ratings for the whole experiment. In this way, the effect of the TMS would necessarily occur during pain processing and we were able to avoid the uncertainty dependent on the interval between painful stimulation and the application of TMS. We expected to see no effect on either pain or distress ratings when applying the TMS on scalp sites correspondent to the caudal MCC and posterior cingulate cortex (in our case, when stimulating Cz and CPz). On the contrary we expected that the stimulation of frontal points (corresponding to Fz, FCz and AFz) would interfere with the antinociceptive and pronoceptive mechanisms mediated by the activity of the ACC or of the anterior part of the MCC.

## Materials and Methods

### Subjects

Thirteen healthy subjects (7 males, 6 females), all right handed (as tested by Oldfield Handedness Questionnaire), took part in the experiment (see Table 1). The experiment consisted of 6 sessions (one per week) of about 1 hour. In each session, participants were subjected to continuous pain for 45 minutes by means of superficial electrical stimulation onto the dominant hand. Subjective ratings of pain (expressed on a 11-point numeric rating scale, NRS, where 0 = no pain and 10 = maximum tolerable pain) and distress (0 = no discomfort and 10 = maximum tolerable distress) were gathered at the start of the experiment and at defined time points (1, 3, 5, 7, 9, 11, 13, 15, 20, 25, 30, 35, 40 and 45^th^ minute). The NRS is widely employed in both clinical and research setting, is a valid and reliable tool to measure the different dimensions of pain [20–21] and has the added benefit of being easy to administer.

**Table 1:** Characteristic of the sample group.

	Mean (SD)
N, M/F	13, 7/6
Age [years]	25 (2)
Depression [HADS]	6 (2)
Distress [DT]	3 (1)
Nasion-Inion distance [cm]	36 (3)
Stimuli intensity [mA]	8.7 (2.7)

M = Males, F = Females, SD = standard deviation, HADS = Hospital Anxiety and Depression Scale, DT = Distress Thermometer

No subject was distressed, depressed or anxious (Table 1), as demonstrated by the scores obtained in the clinical self-reported scales Hospital Anxiety and Depression Scale (HADS, cut-off > 9) and Distress Thermometer (DT, cut-off > 5). No subject reported minor (contusions, tooth- ear- head- or throat-ache) or major pain or any stressful events occurring up to 4 weeks prior or during the study period. The study was conducted in accordance with the Declaration of Helsinki: all subjects gave written consent before participating, and the study was approved by the local ethical committee (Comitato Bioetico d’Ateneo dell’Università di Torino).

### Experimental stimuli

Two pairs of electrodes were positioned on the dominant middle finger and the index finger at a distance of 1 cm. Monophasic, rectangular electrical pulses with duration of 0.5 ms were applied with alternating polarity via a constant current stimulator (Digitimer S7, Digitimer, Hertfordshire, UK) at 6 Hz, with the electrical pulses targeting a different finger every 30 sec. Before the start of the first session we established for each subject the intensity of the current (Table 1), aiming for a pain rating of 5 on the 11-point NRS. This value was used as reference for all subsequent sessions, allowing only small (< 10% of the first session) adjustments to compensate for factors which could have affected the perceived pain. The rating of 5 in the NRS scale was described to the subjects as a stimulation that was clearly painful and in other circumstances would have prompted them to take an analgesic, but could be endured for 45 minutes. No instruction regarding distress ratings was given to participants.

### TMS stimulation

To assess the effect of TMS on the mechanisms of pain adaptation and perception we used a TMS apparatus (MAG&More, München), equipped with a double-cone coil, to stimulate the underlying brain cortex. We applied the stimulation after 5 minutes from the beginning of the painful stimulation. Each participant underwent 6 experimental sessions: a baseline session and five experimental ones. In each experimental session we stimulated one of 5 equidistant points along the medial cortex in antero-posterior direction (10–10 IFCN system [22]: AFz, Fz, FCz, Cz, CPz). The sessions were administered in a pseudorandom order, counterbalanced within subjects. The TMS protocol of stimulation consisted of 5 single pulses, temporally spaced by 30 seconds, at 50%, 70%, 90%, 100% and 100% power fraction of the maximum TMS output, for a total of 2 minutes, in order to achieve the maximum possible depth of stimulation while minimizing the distress for the experimental subject by gradually building up the intensity. The TMS stimuli were biphasic, with the current travelling in the anterior-posterior direction first, and the coil was placed on the scalp in a way so that the handle was kept perpendicular to the skull.

### Data analysis

Data were analyzed using IBM SPSS Statistics Version 21.0 (IBM Corp, Armonk, NY) and Matlab 7.10 (Matworks, Natwick, MA). To assess the habituation effect in the baseline condition we performed a regression (*B* = angular coefficient) using the subjective NRS ratings (pain/distress) as dependent variable and time as independent variable, pooling the data from all subjects. To verify whether TMS stimulation affected the habituation, we first performed a preliminary 2 (time: first and last timepoint) x 6 (location: baseline, AFz, Fz, FCz, Cz, CPz) repeated measure ANOVA. The interaction time by location, if significant, was further explored by means of regression analysis for each location individually.

To assess the immediate effect of TMS on pain and distress, we computed for each session a paired sample Wilcoxon test, comparing the scores gathered before (t = 3 min) and just after (t = 7 min) stimulation. When the test was significant, we calculated the parameter *r* as a measure of the effect size, using the formula *r = |Z/√N|* and using the thresholds of 0.1, 0.3 and 0.5 for a small, medium and large effect size respectively.

Due to the number of comparisons present in this article, we controlled the familywise error rate by applying Finner’s step-down correction [23]. The cutoff for the corrected p value has been set at 0.05.

### ALE meta-analysis

The ALE (Activation Likelihood Estimation) analysis is a quantitative meta-analytic method that can be used to estimate consistent activation across different imaging studies [24]. ALE maps of co-activations are derived based on patterns of foci of interest where multiple studies have reported statistically significant peak activation. Furthermore, it comprises a method to calculate the above chance clustering between experiments (i.e., random effects analysis) rather than between foci (fixed effects analysis).

To obtain a list of neuroimaging papers that investigated pain rating as a variable, we sent a query to the BrainMap database using Sleuth 2.2 [25]. The exact query was:

[Subjects][Diagnosis=Normals]AND[Conditions][External Variable=Pain Rating] (1)

The results of the query were 28 papers (fMRI, PET), 440 subjects, 130 experiments, 1397 foci. We then selected a subset of papers that explicitly calculate a correlation between brain activation and pain ratings. The new subset was composed of 10 papers (fMRI, PET), 195 subjects, 55 experiments, 402 foci. Regions of convergence were calculated using GingerALE 2.3 [24] in the MNI space, correcting for multiple comparisons using the false discovery rate method with no assumption (FDR pN) and the threshold of 0.05 and minimum cluster volume of 1000 mm^3^. A full list of references is in the supplementary materials.

## Results

### Baseline adaptation

**Fig 1:**
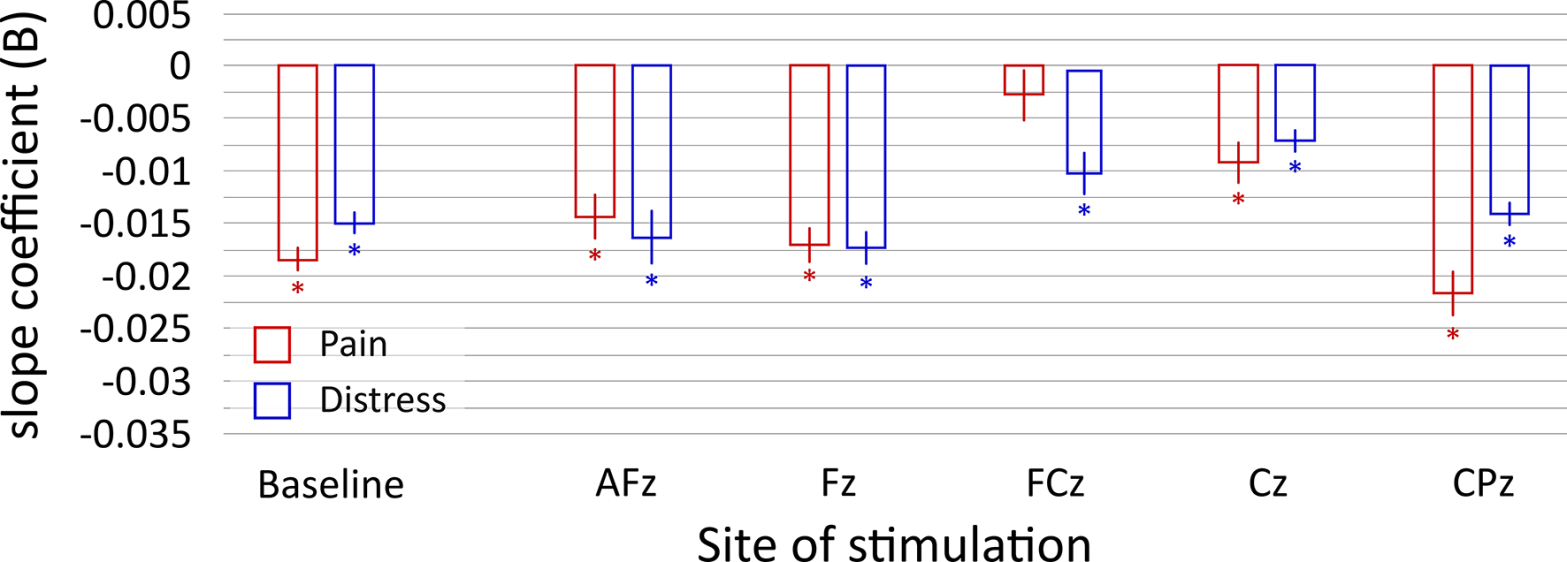
Slope of regression lines for pain and distress ratings, for all stimulation sites and baseline. Asterisks mark significant regressions and error bars represent standard errors.

In the baseline session both perceived pain (*B* = -0.018, *t =* -6.36, *p*_*corr*_ < 0.001) and levels of distress (*B* = -0.015, *t* = -9.59, *p*_*corr*_ < 0.001) decreased linearly with time (Fig 1). Time also explained a significant proportion of variance in both pain (*r* ^*2*^_*corr*_ = 0.753, *F* = 40.52 *p*_*corr*_ < 0.001) and distress scores (*r* ^*2*^_*corr*_ = 0.885, *F* = 92.09, *p*_*corr*_ < 0.001).

### Effect of TMS stimulation on pain ratings

In repeated measure ANOVA, neither the factor *time* (*F* = 3.52, *p*_*corr*_ = 0.11) nor the factor *location* (*F* = 0.16, *p*_*corr*_ = 0.94) were found to be significant, but the interaction *time by location* was significant (*F* = 3.59, *p*_*corr*_ = 0.011).

The regression analysis found that application of TMS on the FCz site inhibited the adaptation mechanism (Fig 1), as perceived pain did not decrease with time (*B* = -0.003, t = -0.87, *p*_*corr*_ = 0.44). On the contrary, application of TMS on the other sites did not inhibit adaptation (AFz *B* = -0.014, t = -3.07, *p*_*corr*_ = 0.017; Fz *B* = -0.017, t = -4.44, *p*_*corr*_ = 0.004; Cz *B* = -0.009, t = -2.58, *p*_*corr*_ = 0.036; CPz *B* = -0.021, t = -7.09, *p*_*corr*_ < 0.001).

**Fig 2:**
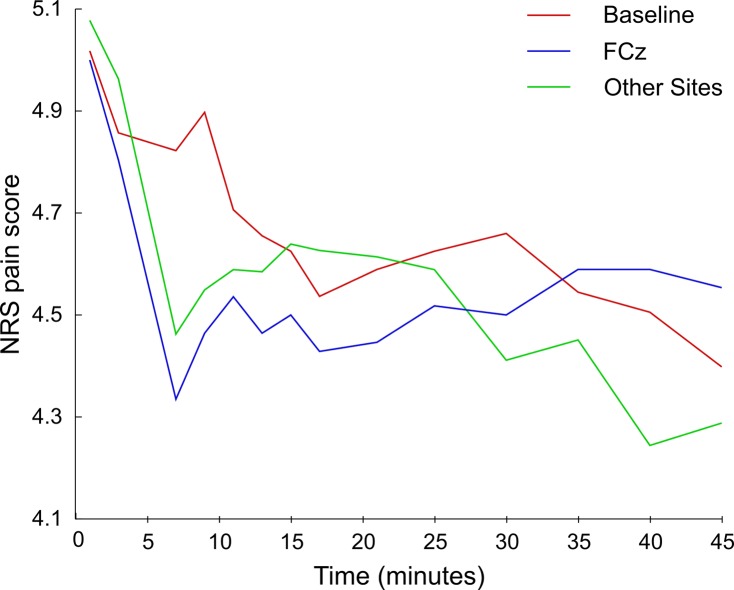
Time series of pain ratings for different experimental sessions: baseline, FCz and other stimulation sites (AFz, Fz, Cz, Cpz).

Pain ratings decreased temporarily immediately after the TMS stimulation, and the drop was significant for all sites: AFz (Z(12) = -2.727, *p*_*corr*_ = 0.012, *r* = 0.75), Fz (Z(12) = -2.829, *p*_*corr*_ = 0.006, *r* = 0.78), FCz (Z(12) = -2.552, *p*_*corr*_ = 0.017, *r* = 0.71), Cz (Z(12) = -2.684, *p*_*corr*_ = 0.011, *r* = 0.74) and CPz (Z(12) = -2.609, *p*_*corr*_ = 0.017, *r* = 0.72, see Fig 2). The average decrease in ratings was of 0.55 points (SD = 0.09), more than it would be expected from the slope of the adaptation effect (0.073) in the baseline session (t(12) = 18.4, *p* < 0.001).

### Effect of TMS stimulation on distress ratings

In repeated measure ANOVA, the factor *time* was found to be significant (*F* = 7.39, *p*_*corr*_ = 0.030) but neither the factor *location* (*F* = 1.15, *p*_*corr*_ = 0.31) or interaction *time by location* (*F* = 0.69, *p*_*corr*_ = 0.75) were significant.

Distress ratings did not change immediately after the TMS stimulation on any site: AFz (Z(12) = -1.86, *p*_*corr*_ = 0.11), Fz (Z(12) = -0.14, *p*_*corr*_ = 0.98), FCz (Z(12) = -1.07, *p*_*corr*_ = 0.39), Cz (Z(12) = -0.84, *p*_*corr*_ = 0.52), CPz (Z(12) = -1,16, *p*_*corr*_ = 0.39).

### ALE meta-analysis

**Fig 3:**
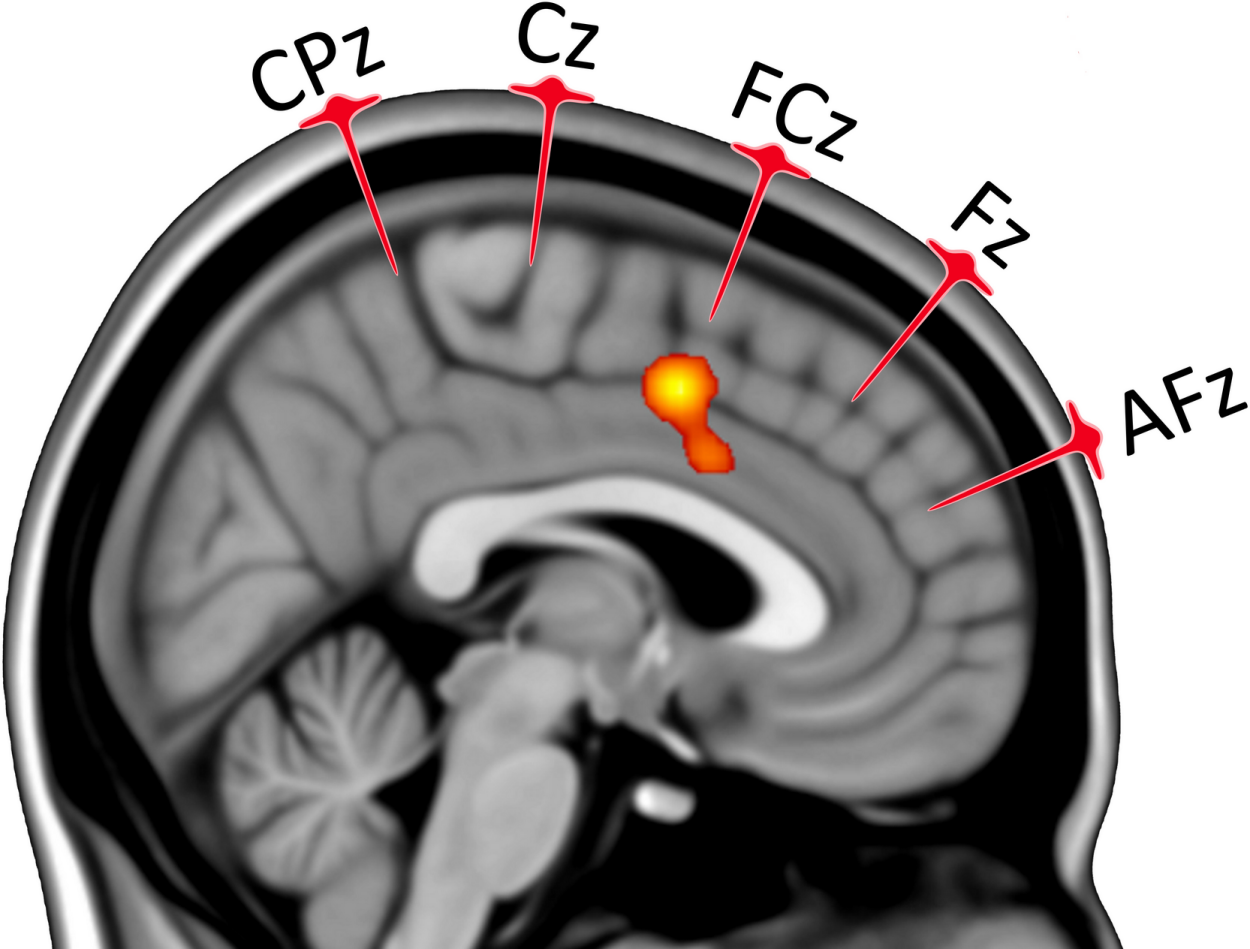
Consistent cingulate ALE cluster, p < 0.05, FDR corrected for multiple comparisons. Ke = cluster extension > 1000 mm^3^. The location of 10–10 EEG medial points AFz, Fz, FCz, Cz and CPz are shown along with their projections. The ALE cluster was under the FCz point, in the MCC. Clusters were overlaid onto a sagittal slice of an MNI atlas (x = 4 mm) using the software Mango, version 3.2.1 (http://ric.uthscsa.edu/mango).

The ALE meta-analysis identified 3 clusters, comprising the right insula (BA 13), right rolandic operculum and ACC (BA 24 and 32, see Fig 3, S1 Fig and S1 Table). The biggest and most significant cluster was localized on the ACC (MNI peak coordinates: x = 4 mm, y = 6 mm, z = 46 mm, Fig 3).

## Discussion

In this paper, we attempted to better clarify the role of the cingulate cortex in adaptation to pain. In order to do so, we stimulated the medial cortex using an interference-like TMS paradigm during continuous painful stimulation. We adopted a novel pain paradigm in which we sought to avoid peripheral habituation effect by alternating the position of the painful stimulation on the hand without avoiding also central habituation [26], as central habituation was the primary mechanism investigated in this work. In order to avoid possible confounds caused by long-term habituation all subjects were tested only once a week, and the absence of long-term habituation was confirmed by the fact that the average starting pain rating was very close to 5 at the beginning of each session.

To be able to stimulate deep brain structures involved in pain processing, such as the cingulate cortex, we used a double-cone TMS coil. This kind of coil has been proven to be able to stimulate deep structures, such as the foot motor cortex and the ACC [13,19] and therefore capable to stimulate the MCC and contiguous brain areas. Furthermore, evidence both from mathematical models and neuroimaging studies [19, 27–28] shows that the MCC can indeed be reached by TMS pulses delivered by a double-cone coil, especially at high intensities like the ones used in this study. It must however be kept in mind that the areas overlying the cingulate cortex were probably stimulated by the magnetic field, as shown by the study of Hayward and colleagues [19], which used H_2_^15^O PET to assess the effect of double-coil TMS stimulation on the ACC and found that while medial frontal TMS using the double-cone coil can modulate the metabolism of the cingulate, contiguous areas were also affected.

During the experiment we gathered both distress and pain ratings at 13 timepoints, using a 0–10 NRS scale. The habituation effect was evident in the baseline condition for both measures, as predicted by previous studies [8,29,30], but it was inhibited, only for pain ratings, after the application of TMS over the FCz site. While the application of the TMS over the other scalp sites (AFz, Fz, Cz, CPz) had no effect on habituation, a significant short-term decrease of pain rating (but not of distress) was found for all five stimulation sites.

To interpret these results it is worth remembering that the rostral cingulate cortex has classically been divided functionally into two areas: the ACC, more involved with affective and emotional tasks [31] and the MCC, involved with cognitive and attentive tasks. A more fine subdivision [32] divides in two areas both the ACC (into sgACC and perigenual ACC, pgACC) and the MCC (anterior and posterior MCC). Theoretically, applying the TMS over the AFz site corresponded to stimulating the pgACC, applying it over the Fz and FCz sites corresponded to stimulating the anterior MCC (aMCC), while TMS over Cz and CPz corresponded to stimulating the posterior MCC, but it must be kept in mind that in the present study the localization of the center of the stimulated area could be determined with less accuracy than in the theoretical case, as we adopted a reference frame of coordinates to administer the TMS pulses instead of neuronavigation.

The immediate effect of TMS on pain ratings regardless of the stimulation site could be either be explained with a generic involvement of the cingulate cortex in the attentional processing or with the distraction caused by the TMS pulses. In particular, orienting the attention away from the painful stimuli has been shown to have analgesic effects [33, 34]. As the analgesic effect was present regardless of the stimulation site, in this study we cannot specifically support one of these two hypotheses.

In this study we did not see an effect of TMS on distress ratings. This was not expected, but can be explained by the fact that our experimental setup allowed partial stimulation of the pgACC and did not allow at all the stimulation of the sgACC, which has been more strongly linked to emotional and affective tasks [35].

An explanation of the MCC causal role can be found in the model of Shackman [36], which replaces the idea of functional segregation in the cingulate cortex with the integration of pain processing, negative affect and cognitive control in the aMCC, which could be appropriately described as a neural hub. In this framework, the MCC would then serve a general high role in pain processing. The idea that specific pain processing functions are not segregated in the different parts of the cingulate cortex is further supported by the somehow contradictory effects reported in literature by stimulating anterior and posterior areas. In fact, contiguous areas can have apparently very different roles, as, for instance, noted by [37], whose meta-analysis of the placebo effect found that the ACC metabolism was positively correlated to the placebo analgesia while the middle and posterior cingulate cortex metabolism was negatively correlated.

Our results were in agreement with the model of Shackman and colleagues as the suppression of habituation of pain was found when applying the TMS on the FCz site. This site corresponded to stimulating the MCC and the area of activation defined by our ALE analysis both lies within the hub evidenced by the conjunction analysis of Shackman and colleagues and precisely corresponds to the FCz stimulation site (Fig 3).

The ALE meta-analysis included in this work also shows with greater spatial accuracy that applying double-cone coil TMS over the FCz site allows stimulation of an area that is implicated in the subjective rating of pain, bringing evidence gathered from neuroimaging studies. We can therefore argue that the effect we observed in our paper was likely to be due to the stimulation of MCC rather than of contiguous areas affected by the induced electric fields.

In fact, using the TMS as methodology we were able to directly support the causal role of the aMCC in the central habituation to pain. This has been observed in correlational studies, such as the ones investigating pain habituation mechanisms in migraine patients [38,39]. These studies found an alteration of laser evoked potentials (LEPs, whose origin, in the case of N2 and P2, has been suggested to be in the MCC [40,41]) along with a decreased habituation to pain, while another study [42] confirmed the LEPs neural localization to be inside the MCC (called ACC in the paper). In conclusion, the present work confirms the role of the cingulate cortex in habituation, demonstrates the causality of this region in the process and better specifies the hotspot of the area involved.

## Supporting Information

S1 Fig Areas correlated to subjective pain ratings. Consistent ALE clusters, p < 0.05, FDR corrected for multiple comparisons. Ke = cluster extension > 1000 mm^3^. Left to right sagittal slices. Brain ALE clusters were overlaid onto an MNI atlas using the software Mango, version 3.2.1 (http://ric.uthscsa.edu/mango).
(JPG)

S1 Table Coordinates of areas correlated to subjective pain ratings. Coordinates of peaks of Consistent ALE clusters, p < 0.05, FDR corrected for multiple comparisons. Ke = cluster extension > 1000 mm3. (BA) = Brodmann Area. L = Left. R = Right. x, y, z expressed in mm. Coordinates were reported in MNI space. Brain regions were classified using Talairach Daemon Tool (http://www.talairach.org/daemon.html).
(DOC)
