# Supplementary figures and images for: Double-Cone Coil TMS Stimulation of the Medial Cortex Inhibits Central Pain Habituation
Source: PLoS One. 2015 Jun 5;10(6):e0128765. doi: 10.1371/journal.pone.0128765 (PMC4457929; doi:10.1371/journal.pone.0128765)

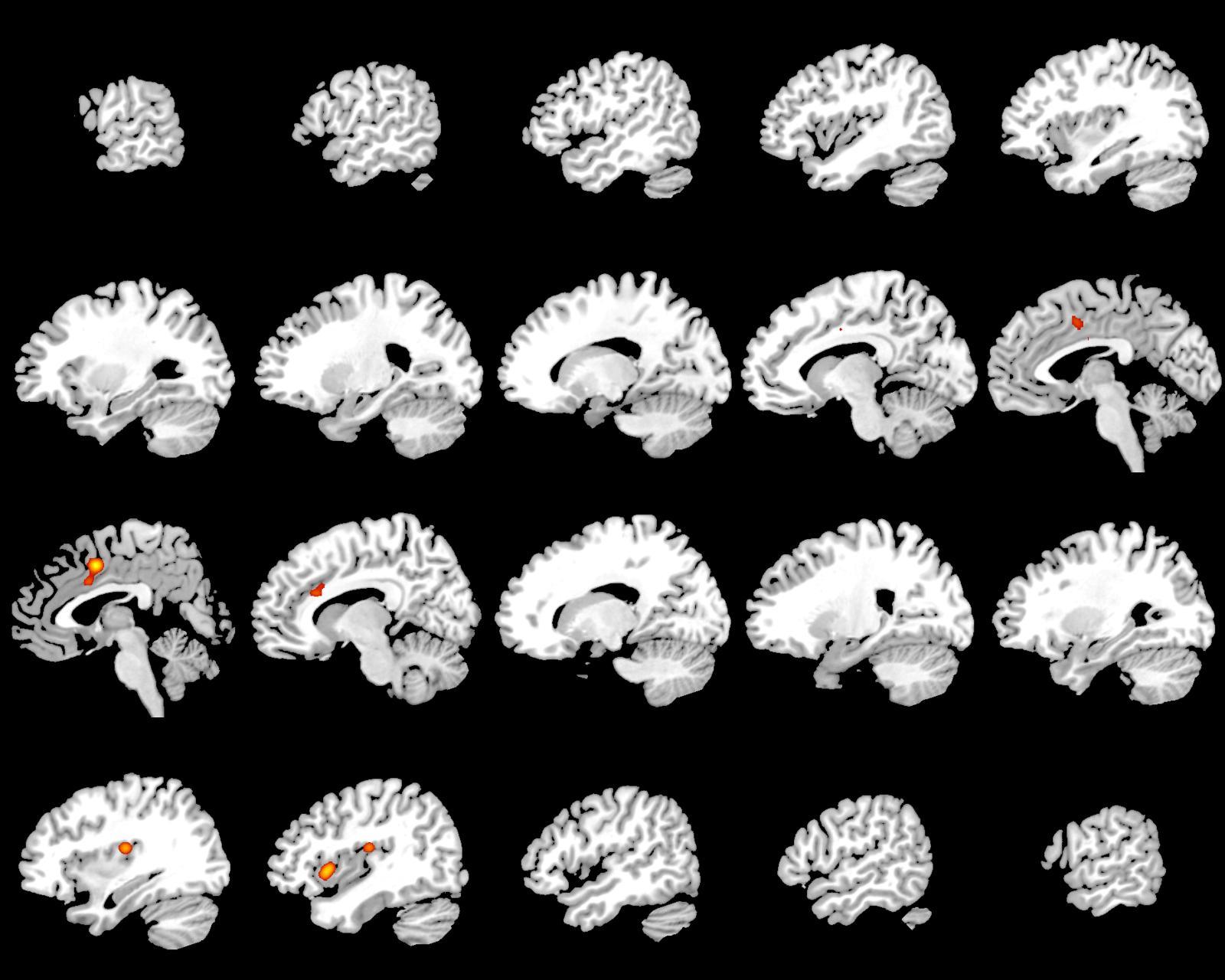

Supplement: S1 Fig — Consistent ALE clusters, p < 0.05, FDR corrected for multiple comparisons. Ke = cluster extension > 1000 mm3. Left to right sagittal slices. Brain ALE clusters were overlaid onto an MNI atlas using the software Mango, version 3.2.1 (http://ric.uthscsa.edu/mango). (JPG) [file pone.0128765.s001.jpg]
