# Supplementary material for: Double-Cone Coil TMS Stimulation of the Medial Cortex Inhibits Central Pain Habituation
Source: PLoS One. 2015 Jun 5;10(6):e0128765. doi: 10.1371/journal.pone.0128765 (PMC4457929; doi:10.1371/journal.pone.0128765)
Supplement: S1 Table — Coordinates of peaks of Consistent ALE clusters, p < 0.05, FDR corrected for multiple comparisons. Ke = cluster extension > 1000 mm3. (BA) = Brodmann Area. L = Left. R = Right. x, y, z expressed in mm. Coordinates were reported in MNI space. Brain regions were classified using Talairach Daemon Tool (http://www.talairach.org/daemon.html). (DOC) [file pone.0128765.s002.doc]

**Table S2. Coordinates of areas correlated to subjective pain ratings**

| **Cluster** | **Brain Region** | **mm^3^** | **x** | **y** | **z** | **Max ALE** |
| --- | --- | --- | --- | --- | --- | --- |
| 1 | R Cingulate Gyrus (BA 24) | 3496 | 4 | 6 | 46 | 0.020 |
|  | R Cingulate Gyrus (BA 24) |  | 8 | 24 | 24 | 0.013 |
|  | R Cingulate Gyrus (BA 24) |  | 2 | 10 | 32 | 0.012 |
|  | R Cingulate Gyrus (BA 32) |  | 8 | 20 | 30 | 0.010 |
|  | R Cingulate Gyrus (BA 24) |  | -8 | -2 | 38 | 0.010 |
|  | R Cingulate Gyrus (BA 24) |  | -6 | -4 | 32 | 0.009 |
| 2 | R Insula (BA 13) | 1184 | 42 | 16 | -2 | 0.018 |
| 3 | R Rolandic Operculum | 1088 | 38 | -16 | 16 | 0.018 |

Coordinates of peaks of consistent ALE clusters, p < 0.05, FDR corrected for multiple comparisons. Ke = cluster extension > 1000 mm^3^. (BA) = Brodmann Area. L = Left. R = Right. x, y, z expressed in mm. Coordinates were reported in MNI space. Brain regions were classified using Talairach Daemon Tool (www.talairach.org/daemon.html)

**Meta-analysis references**

1. Christmann C, Koeppe C, Braus D. F, Ruf M, Flor H (2007) A simultaneous EEG-fMRI study of painful electric stimulation. NeuroImage 34: 1428–1437.

2. Geuze E, Westenberg H. G. M, Jochims A, de Kloet C. S, Bohus M, Vermetten E, Schmahl C (2007) Altered pain processing in veterans with posttraumatic stress disorder. Archives of General Psychiatry 64: 76–85.

3. Derbyshire S. W. G, Jones A. K. P, Creed F, Starz T, Meltzer C. C, Townsend D. W, Peterson A. M, Firestone L. L (2002) Cerebral responses to noxious thermal stimulation in chronic low back pain patients and normal controls. NeuroImage 16: 158–168.

4. Cheng Y, Lin C. P, Liu H. L, Hsu Y. Y, Lim K. E, Hung D, Decety J (2007) Expertise modulates the perception of pain in others. Current Biology 17: 1708–1713.

5. Guo X, Zheng L, Wang H, Zhu L, Li J, Wang Q. D, Dienes Z, Yang Z (2013) Exposure to violence reduces empathetic responses to other’s pain. Brain and Cognition 82: 187–191.

6. Derbyshire S. W. G, Nichols T. E, Firestone L. L, Townsend D. W, Jones A. K. P (2002) Gender differences in patterns of cerebral activation during equal experience of painful laser stimulation. Journal of Pain 3: 401–411.

7. Coan J. A, Schaefer H. S, Davidson R. J (2006) Lending a hand: Social regulation of the neural response to threat. Psychological Science 17: 1032–1039.

8. Tolle T. R, Kaufmann T, Siessmeier T, Lautenbacher S, Berthele A, Munz F, Zieglgansberger W, Willoch F, Schwaiger M, Conrad B, Bartenstein P (1999) Region-specific encoding of sensory and affective components of pain in the human brain: A positron emission tomography correlation analysis. Annals of Neurology 45: 40–47.

9. Obermann M, Pleger B, de Greiff A, Stude P, Kaube H, Diener H. C, Katsarava Z (2009) Temporal summation of trigeminal pain in human anterior cingulate cortex. NeuroImage 46: 193–200.

10. Kong J, White N. S, Kwong K. K, Vangel M. G, Rosman I. S, Gracely R. H, Gollub R. L (2006) Using fMRI to disssociate sensory encoding from cognitive evaluation of heat pain intensity. Human Brain Mapping 27: 715–721.
